# Bone Regeneration Capabilities of Scaffolds Containing Chitosan and Nanometric Hydroxyapatite—Systematic Review Based on In Vivo Examinations

**DOI:** 10.3390/biomimetics9080503

**Published:** 2024-08-20

**Authors:** Paweł J. Piszko, Aleksandra Piszko, Sylwia Kiryk, Jan Kiryk, Tomasz Horodniczy, Natalia Struzik, Kamila Wiśniewska, Jacek Matys, Maciej Dobrzyński

**Affiliations:** 1 Department of Pediatric Dentistry and Preclinical Dentistry, Wroclaw Medical University, Krakowska 26, 50-425 Wrocław, Poland; pawel.piszko@umw.edu.pl (P.J.P.);; 2 Department of Dental Surgery, Wroclaw Medical University, Krakowska 26, 50-425 Wrocław, Poland; 3 Ortho.pl Centrum Zdrowego Uśmiechu, Buforowa 34, 52-131 Wrocław, Poland; 4 Pre-Clinical Research Centre, Wroclaw Medical University, Bujwida 44, 50-368 Wrocław, Poland

**Keywords:** bone substitute, bone tissue, chitosan, graft material, hydroxyapatite

## Abstract

In this systematic review, the authors aimed to investigate the state of knowledge on in vivo evaluations of chitosan and nanometric hydroxyapatite (nanohydroxyapatite, nHAp) scaffolds for bone-tissue regeneration. In March 2024, an electronic search was systematically conducted across the PubMed, Cochrane, and Web of Science databases using the keywords (hydroxyapatite) AND (chitosan) AND (scaffold) AND (biomimetic). Methodologically, the systematic review followed the PRISMA (Preferred Reporting Items for Systematic Reviews and Meta-Analyses) protocol to the letter. Initially, a total of 375 studies were screened, and 164 duplicates were removed. A further 188 articles were excluded because they did not correspond to the predefined topics, and an additional 3 articles were eliminated due to the inability to obtain the full text. The final compilation included 20 studies. All publications indicated a potential beneficial effect of the scaffolds in in vivo bone defect repair. A beneficial effect of hydroxyapatite as a scaffold component was observed in 16 studies, including greater mechanical resistance, cellular differentiation, and enhanced bone damage regeneration. The addition of chitosan and apatite ceramics, which combined the strengths of both materials, had the potential to become a useful bone-tissue engineering material.

## 1. Introduction

Bone is a metabolically active tissue that undergoes constant rebuilding and remodeling throughout the lifespan of an individual [1]. This implies that it has the capacity for regeneration, which can occur following injury [2,3]. However, as the organism ages or is depleted of vital substrates, this ability is diminished, or the process becomes imbalanced [4]. Furthermore, natural processes are inadequate in cases of larger bone defects. The critical size threshold for a bone defect is approximately 2 cm or greater than 50% loss of the circumference of the bone. A tissue deficit may result in nonunion, malunion, or pathological fracture [5]. In such situations, grafting materials become immensely useful. Nevertheless, allogeneic, xenogeneic, and autologous materials exhibit relatively high degradation rates, with a concomitant reduction in bioactivity over time. One of the objectives of bone-tissue engineering is to develop functional alternatives that induce osteoconduction, provide mechanical stability, and integrate into the bone structure [6].

**Figure 1:**
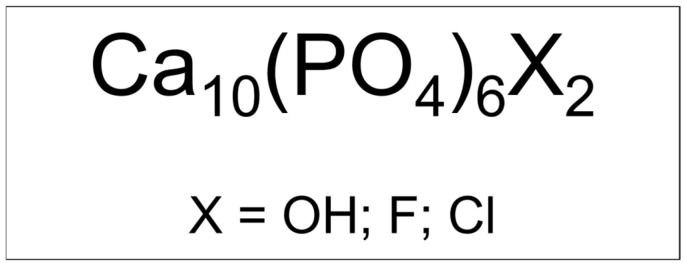
Possibilities of structural modifications of HAp.

Apatite is a ubiquitous mineral found in the rocks of the Earth, the soil of the Moon, meteorites from Mars, and the hard tissues of vertebrates [7]. Apatite structures have the general formula of Ca_10_(PO_4_)_6_X_2_, where X can most commonly be substituted by a hydroxyl group, fluoride, or chlorine (Figure 1) [8]. Apatite structures are very versatile and can be chemically modified. In the context of apatite ceramics, this affects their solubility, hardness, brittleness, strain, thermal stability, and optical properties such as birefringence [9], making apatite materials a useful substance in bone-tissue engineering. Among various apatite compounds present within the human body, hydroxyapatite (HAp), which contains hydroxyl groups, is the most prevalent. It is capable of forming a direct chemical bond with surrounding tissues, has osteoconductive properties, and is non-toxic, non-inflammatory, and non-immunogenic [10]. In addition to its application in the medical field, where it is employed in the repair and regeneration of bone and teeth, hydroxyapatite has a number of applications in the industrial sector. These include the production of fertilizers and pharmaceutical products, protein chromatography, and water treatment processes [11]. Incorporating apatite ceramics into bone-tissue engineering materials is a popular practice today. Its porosity is highly desirable, and it creates a space for cells to migrate [7]. Its similarity to natural bone makes the material highly compatible [12]. Nevertheless, the utilization of pure HAp is currently constrained by its unfavorable brittleness [11]. Consequently, novel composite materials have been developed with the objective of accentuating the benefits of HAp while simultaneously mitigating its inherent limitations.

Bone tissue can be defined as a nanocomposite, predominantly comprising nanohydroxyapatite and type-I collagen [13]. This is the rationale behind the extensive utilization of nanohydroxyapatite in grafting materials. Nanoparticles constitute a class of materials that display unique properties compared to their bulk and molecular counterparts. They have the capacity to elicit a biological response and augment mechanical properties [14]. The nano form of the materials has been demonstrated to promote the differentiation of bone-marrow-derived mesenchymal stem cells into osteoblasts, with this process being selectively triggered by integrin receptors [15]. A number of techniques may be employed in the synthesis of nanohydroxyapatite. These include the preparation of nanohydroxyapatite powder in a solid-state reaction, sol–gel methods, hydrothermal route and co-precipitation methods, mechano-chemical method, microwave irradiation, and ultrasonic-assisted process [16,17].

**Figure 2:**
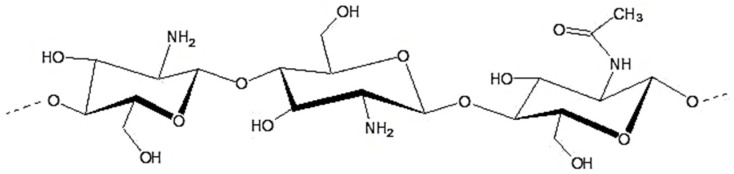
Outlook on structure of chitosan polymeric chain.

Biopolymers, otherwise known as natural polymers, constitute a group of macromolecules that are formed under natural conditions by living organisms. These polymers are distinguished by their high degree of stability. The most widely known examples of biopolymers are cellulose and starch. Nevertheless, there is a growing interest in more intricate hydrocarbon polymers produced by bacteria and fungi, particularly polysaccharides such as xanthan, curdlan, pullulan, chitin, chitosan, and hyaluronic acid [18]. Chitosan represents a notable exception within this group, offering a range of advantageous properties that align with those required in medical applications. These include biocompatibility, biodegradability, mucoadhesion, anticholesterolemic, hemostatic, antimicrobial, and even antitumoral effects [19], which are not exhibited by other materials from this group at all, or not to the same extent. Chitosan is a natural polymer derived from chitin—the second most abundant biopolymer in nature after cellulose. Chitin is found in crustaceans, insects, and fungi [20]. The monomer of chitosan is β-1,4-D-glucosamine, which differs only slightly from the monomer of chitin, N-acetyl-D-glucosamine, by the absence of the acetyl group [20] (Figure 2). While the use of chitin is quite limited due to its insolubility and intractable nature, chitosan has much better properties and can, therefore, be used for a variety of purposes. It has a better solubility profile, less crystallinity, and is amenable to chemical modification due to the presence of functional groups such as hydroxyl, acetamido, and amine [21]. Chitosan is widely used in the field of medicine due to its many desirable properties. It has been reported that chitosan is used as a wound dressing, a material for repairing broken nerve endings, a carrier for various volume-expanding or slow-release drugs, an anti-tumor remedy, and a material for bone or cartilage engineering [22]. The combination of chitosan and apatite ceramics, intertwining both their strengths, has a great potential for becoming a useful bone- and cartilage-tissue engineering material [23]. The composite can act as a material with the potential to facilitate multi-lineage stem-cell differentiation and even localized gene delivery [24,25]. Inquiry into how to further enhance, by modifications or additions, such an organic–nonorganic union is even more essential for developing a new reliable bone deficit filler [26]. A summary and an evaluation of the topic are needed, as it could set the path for future research.

## 2. Materials and Methods

### 2.1. Focused Question

**Figure 3:**
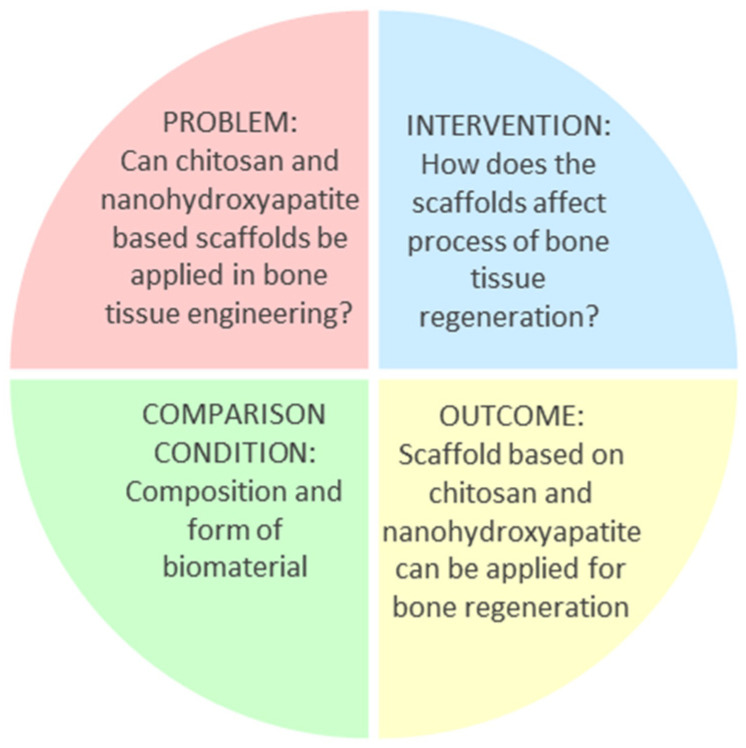
PICO framework of the presented study.

The systematic review followed the PICO (Population/Patient/Problem; Intervention; Comparison; Outcome) framework [27], as follows (Figure 3):

The PICO question is: among scaffolds containing chitosan and nanohydroxyapatite (population), do in vivo tests (investigated condition) showcase enhanced bone-tissue regeneration (outcome) compared to different compositions and forms of biomaterial (comparison condition)?

### 2.2. Protocol

**Figure 4:**
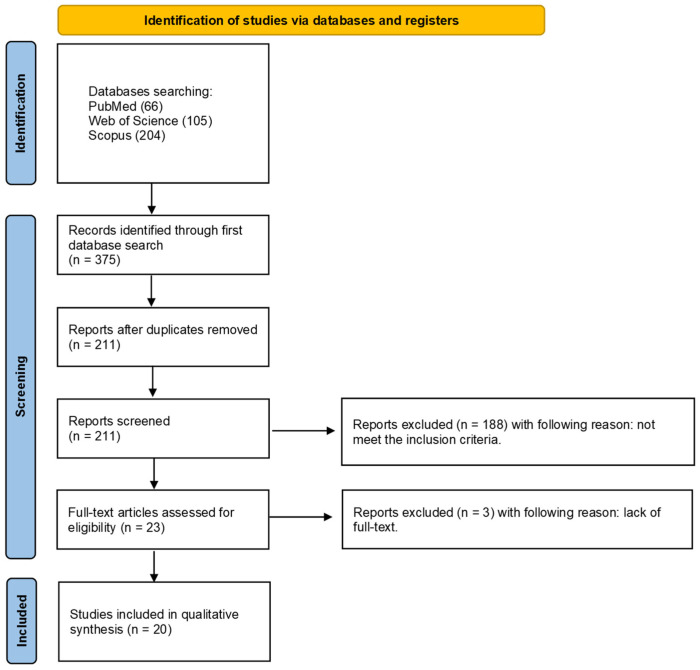
PRISMA 2020 flow diagram of the presented study.

The article selection process for the systematic review was outlined carefully, following the PRISMA flow diagram [28] and presented in Figure 4. The systematic review was registered on the Open Science Framework (OSF) under the following address: https://doi.org/10.17605/OSF.IO/69W8Y (access date: 6 June 2024).

### 2.3. Eligibility Criteria

All studies included in the systematic review were required to meet specific criteria, including the investigation of scaffolds containing chitosan and nanohydroxyapatite in their matrix in the scope of bone-tissue regeneration, in vivo examination, and publication in the English language. The reviewers collectively established exclusion criteria, which included studies published in languages other than English, clinical reports, opinions, editorial papers, review articles, and studies lacking a full-text version. During the database search, it was noted that, although authors utilize nanohydroxyapatite in their studies, they tend to refer to it generally as hydroxyapatite. Therefore, articles were manually screened to verify the structure of HAp and include only those studies in which it was present in the nanometric form (at least one of the dimensions is nanometrically sized) [29]. Hence, the search string presented in Section 2.4. included (hydroxyapatite) instead of (nanohydroxyapatite).

### 2.4. Information on Sources, Study Selection, and Search Strategy

A search was conducted in March 2024 using the PubMed, Scopus, and Web of Science (WoS) databases. In the Scopus database, the search results were refined to include only titles, abstracts, and keywords. In PubMed, they were limited to titles and abstracts. In WoS, the results were restricted solely to abstracts. The search criteria were based on the following keywords: (hydroxyapatite) AND (chitosan) AND (scaffold) AND (biomimetic). All searches were conducted in accordance with the established eligibility criteria, and only articles with available full-text versions were considered.

### 2.5. Data Collection and Data Items

The selected articles were evaluated to ascertain whether they met the predefined criteria. Subsequently, the data were collated and organized in a standardized format.

### 2.6. Assessing the Risk of Bias of Individual Studies

At the stage of study selection, the titles and abstracts of each study were independently checked by the authors to minimize the potential for reviewer bias. The level of agreement among the reviewers was determined using the Cohen κ test. Any discrepancies in opinion regarding the inclusion or exclusion of a study were resolved through discussion between the authors. The risk of bias was evaluated based on the quality assessment. 

### 2.7. Quality Assessment (QA)

Two independent evaluators (P.J.P. and T.H.) conducted a systematic appraisal of the procedural quality of each study within the article. The assessment criteria were selected to focus on critical information regarding the use of scaffolds containing chitosan and nanohydroxyapatite. In evaluating the study design, implementation, and analysis, criteria were applied, including a minimum in vivo test-group size of 10 animals, the presence of a control group in in vivo examination, a detailed description of the biomaterial composition used in the study, a description of the effect of the scaffold on the process of bone regeneration, and a description of the potential clinical applicability of the biomaterial. The studies were assigned points on a scale of 0 to 5, with a higher score indicating superior study quality and a lower risk of bias. The evaluation of the risk of bias was conducted according to the following scoring system. A score of 0–1 indicates a high risk, 2–3 denotes a moderate risk, and 4–5 signifies a low risk. Any discrepancies in the scoring were resolved through a comprehensive discussion until a consensus was reached [27,30,31,32,33,34,35].

## 3. Results

### 3.1. Study Selection

The initial database search across PubMed, Scopus, and WoS yielded 375 articles that were potentially relevant for the presented review. Following the removal of duplicates, 211 articles underwent screening. The initial screening of titles and abstracts resulted in the exclusion of 188 articles that did not involve a comparison of analysis between different specialists. Subsequently, 23 articles underwent further full-text analysis, during which 3 articles were excluded for not meeting the inclusion criteria. In conclusion, a total of 20 articles were included in this review. The considerable heterogeneity among the included studies precludes the possibility of conducting a meta-analysis.

### 3.2. General Characteristics of the Included Studies

The studies included in the systematic review present the influence of scaffolds containing at least chitosan and nanohydroxyapatite on in vivo bone-tissue regeneration. The general characteristics of the studies are presented in Table 1. A total of eleven studies employed rats as subjects for in vivo examinations, while four studies used rabbits. Five of the studies were conducted on mice. The techniques employed in the manufacture of scaffolds included co-precipitation, freeze gelation, and biomimetic processes. The authors frequently utilized the freeze-drying method. All of the studies concluded that the evaluated biomaterials may be successfully used in bone-tissue engineering.

### 3.3. Main Study Outcomes

A detailed characterization of the selected studies is presented in Table 2. The objective of this review was to compare and assess studies concerning scaffolds made primarily of chitosan and apatite. In the majority of studies [36,37,38,39,41,42,44,45,46,47,50,51,52,53], the primary focus of the examination of said scaffolds was their use in regeneration and new bone formation. Three groups of researchers approached the subject from a broader perspective, examining the use of CS/HA scaffolds in bone-tissue engineering in general [44,54,55]. Korpayev’s approach [40] involved the development of multi-layered osteochondral mimetic constructs without the need for growth factors, with the conclusion of the research suggesting a potentially higher elastic modulus. An innovative approach can be found in the studies of Huang et al. [43]. Researchers composed and examined a minimally invasive injectable biomimetic gel scaffold that provides a biocompatible environment for bone-marrow stem cell survival and can serve as a cell carrier. A similar approach regarding hydrogels in tissue engineering was employed by Ju et al. [56]. Good vascularization is essential for uninterrupted bone regeneration, and neovascularization is an important parameter in bone regeneration. Yu et al. [49] achieved notable effects by incorporating copper ions into the scaffold.

The composition of the materials examined differed throughout the articles analyzed. Two studies by Korpayev et al. [41] and Kong et al. [54] focused on scaffolds made solely of pure chitosan and hydroxyapatite. Furthermore, Korpayev et al. [40] did not assess a reference sample; instead, they focused on the different properties of the various layers of the material. In the other article, the main substrates have been either enhanced or an additional component has been added to the mixture. In two articles [36,38], the chitosan phase was subjected to carboxymethylation. In turn, hydroxyapatite has been modified in three studies by the addition of lanthanum [37], zinc [47], and copper [48]. In addition to the aforementioned substrates, other materials were employed in the studies, including B-cyclodextrin [36], icariin [39,42], genipin [41], and collagen [38,40,43,44,50]. Furthermore, others employed poly (lactic-co-glycolic acid) [45], Fe_3_O_4_ [46], BMP2-derived peptide [44], copper [49], and silk fibroin [51]. Other materials that have been investigated include sodium carboxymethyl cellulose [52], bone-marrow mesenchymal stem cells [53], and hyaluronic acid oligosaccharides [55].

The biomimetic nature of the scaffolds provides appropriate conditions for bone reconstruction, as indicated in studies [36,37,41,46,47,51]. The porous structure and physical and chemical properties comparable to human tissue provide an environment for the formation of new bone. A study by Chen et al. found that the evaluated materials effectively initiated bone repair processes in vivo [44]. These processes showcase the biomimetic nature of the CS/HAp scaffolds and their ability to promote in vivo bone-tissue regeneration.

Six studies [36,37,43,44,50,54] did not investigate hydroxyapatite particles and instead focused on the overall influence and performance of the implanted scaffold. Those researchers who focused on HA unanimously attest that hydroxyapatite and its modifications enhance the properties of the scaffolds. It is said to enhance alkaline phosphatase activity [46], mechanical properties [39,47], bioactivity [39], cellular infiltration and activity [44,53,55], and osseointegrative capability [41]. Furthermore, it can facilitate the resolution of inflammation [46]. As stated by Yu [49], the morphology of HA particles renders them suitable for use as drug-delivery vessels. Furthermore, as demonstrated by Lai [51], hydroxyapatite has the potential to facilitate the differentiation of bone-marrow stem cells. Lai [51] additionally posited that the impact of HA is contingent upon its concentration within the scaffold. It was also demonstrated that modified hydroxyapatite can be an effective addition to the scaffold. Frohbergh [41] reports delayed degradation profiles when adding HA, whereas Chen et al. [44] state that HA facilitates said degradation. This discrepancy represents a notable inconsistency identified by the authors of this review, and it is a topic that merits further investigation.

Twelve of the selected studies included an evaluation of the mechanical properties of the scaffolds [36,37,39,40,42,44,45,46,47,49,51,55]. Jolly et al. reported that a BCHD3 scaffold containing carboxymethyl chitosan, nanohydroxyapatite, β-cyclodextrin, and date-seed extract exhibited a compressive modulus of 1533 MPa, which corresponds to the mechanical properties of human cortical bone [36]. The scaffold reported by Yin et al. (La-doped CS/nHAp) matched the mechanical strength of trabecular bone [37]. Hu et al. [39] put an emphasis on the increase of compressive strength of the scaffold with a greater addition of HAp (up to 3 wt.%). Korpayevs’ study reported a gradient increase in compressive strength for a multilayer scaffold, indicating the regeneration of osteochondral defects [40]. Wu et al. [42] stated that the addition of icariin to the CS/HAp scaffold decreased the elastic modulus and fracture strength, but icariin itself boosted the scaffolds’ osteoconductive and osteoinductive potentials. On the other hand, the addition of Fe_3_O_4_ to the CS/HAp matrix enhanced compressive resistance [46], while the doping of HAp microspheres with Cu did not affect the compressive mechanical properties significantly [49]. Subsequent studies indicated that the addition of HAp to the polymer-based matrix increased compressive strength [44,45,47,55]. This conclusion is persistent throughout the studies evaluated in the presented systematic review. Finally, Lai et al. [51] implied that the homogenous incorporation of apatite ceramics into the matrix enhances mechanical stability. Therefore, side-by-side morphological and mechanical assessment is beneficial for concluding the relation between the structure and its properties.

### 3.4. Quality Assessment

Among the 20 articles included in the review, 1 was assigned with a high risk of bias [43], 2 with a moderate risk of bias [40,55], and the remaining 17 with a low risk of bias [36,37,38,39,41,42,45,46,47,48,49,50,51,52,53,54,57] (see Table 3).

## 4. Discussion

This review discussed research relevant to the use of scaffolds containing chitosan, hydroxyapatite, and other compounds that enhance their properties for bone-tissue regeneration. The current engineering technologies enable the construction of synthetic, complex biomaterials that exhibit properties nearly as good as those of natural bone and are highly biocompatible. In cases of tooth loss and subsequent alveolar atrophy, bone-substitute materials play a significant role in the treatment process. Composite grafts offer the advantages of autografts and allografts in a single surgical procedure, combining the best of both techniques. Such a graft may be constructed by combining a synthetic scaffold with biological elements, thereby stimulating cell infiltration and new bone formation [58]. The rising incidence of craniofacial cancer further contributes to the formation of critical bone defects. Although allogeneic, xenogeneic, and autogenous materials have been used for years to treat patients, there is a need to develop new materials to address the increasing challenges faced by clinicians in treating bone defects. The number of articles selected for this review concerning materials based on chitosan–hydroxyapatite scaffolds for in vivo bone regeneration is limited, indicating a need for further extensive research. Chitosan-based scaffolds and their modifications allow for the treatment of increasingly larger and more complex bone defects without causing complications and therapeutic failures.

Biomimetic bone-substitute materials are characterized by a morphology and physical and chemical properties similar to human bone. After application, the material takes over the function of the autologous bone, enabling the regeneration of the defect. These materials are intended to resemble natural bone as much as possible. As mentioned previously, HAp used in scaffolds is also a component of natural bone. Research conducted by Jolly et al. [36] and Huang et al. [43] show that scaffolds containing HAp have a biomimetic character and proadhesive properties. They caused an osteogenic effect and revascularization of the newly formed bone, which suggests a high biomimetic potential. A histological analysis of the collected samples showed a high degree of resorption and replacement of the bone-substitute material with newly formed bone. The lack of immunological reaction and inflammation, caused by the significant similarity of the material to natural bone, additionally emphasizes the biomimetic potential of the scaffolds.

The structure and morphology of scaffolds are important, as noted by Kong et al. [54] in their research. The multilayer nanohydroxyapatite–chitosan scaffold, compared to the uniform nanohydroxyapatite/chitosan scaffold, has much larger pores and leads to greater biocompatibility, prevents the formation of fibrous tissue in the defect, and ensures better delivery of nutrients to the newly formed bone. In the analyzed studies, the average scaffold structure had a porous structure, with an average pore size ranging from 30–90 μm [48] to even 200–400 μm in research by Sun et al. [47].

For several years, the use of autogenous materials necessitated the collection of material from another site, posing a potential risk of complications at the donor site. Currently, widely available bone-substitute materials serve as good alternatives for treating patients needing bone defect reconstruction. Their use not only reduces the risk of complications but also significantly accelerates and improves the procedure. Detailed characterization of these materials allows for better prediction of possible complications and the implementation of appropriate treatment regimens at the onset of symptoms.

Surface-functionalized, controlled/sustained release, preprogrammed release, stimuli-responsive, and gene delivery are the five main categories of biomolecule delivery platforms [59]. The role of growth factors (GFs) in the bone repair process has been extensively documented [60]. There have been numerous studies on the effects of growth factors in enhancing the in vitro chondrogenic potential and in vivo cartilage regeneration [61,62,63,64]. The utilization of growth factors is also employed in the regeneration of bone tissue [63,65,66]. Scaffolds based on chitosan and hydroxyapatite allow for the delivery and controlled release of drugs, as shown by Zeng et al. [67] in their research. This enables the adoption of new strategies for the treatment of critical bone defects and chronic bone disease processes. In vivo, animal studies demonstrated the biocompatibility of the obtained scaffolds and the osteoconductive effect. This produced significantly better results in the treatment of bone defects compared to the control group. The physical properties of the scaffolds allowed for obtaining a stable structure for osteoblasts and mineral components of the newly formed bone. The objective of utilizing substitute materials is to facilitate biological integration while stimulating the body’s intrinsic capacity for tissue regeneration.

Most of the analyzed articles were published in journals related to chemistry and biomaterials. It is appropriate to widely disseminate knowledge about biomaterials used to regenerate bone defects. This approach may increase interest in this topic among doctors and researchers who deal with the treatment of bone defects that exceed the regenerative capabilities of the human body on a daily basis. The increasing role of regenerative methods is worth mentioning in the context of dentistry, especially dental surgery. This field encompasses the repair, restoration, and replacement of defective or non-functional tissues that have been lost due to various diseases, regressive changes, congenital defects, or damage.

## 5. Conclusions

This systematic review outlines the promising advances in scaffolds containing chitosan, nanohydroxyapatite, and other compounds for bone-tissue regeneration application. The integration of these materials into biomaterials has resulted in highly biocompatible and effective substitutes for natural bone, offering significant benefits for the treatment of bone defects, based on in vivo examinations. The review highlights the diverse approaches and materials utilized in the development of chitosan- and apatite-based scaffolds for bone regeneration. The studies examined illustrate a broad spectrum of innovations, including the creation of multi-layered osteochondral mimetic constructs, cost-effective bioactive composites, and injectable biomimetic gels. Modifications to both chitosan and hydroxyapatite, including carboxymethylation, phosphorylation, and the addition of various ions and polymers, have been shown to enhance scaffold properties, such as mechanical strength, bioactivity, and cellular infiltration. It is notable that the integration of hydroxyapatite generally improves the performance of the scaffold. However, discrepancies in degradation rates suggest that further investigation is required. The mechanical properties of the scaffolds have been subjected to rigorous evaluation, with numerous studies corroborating the assertion that the incorporation of hydroxyapatite markedly augments compressive strength. These findings emphasize the necessity of a comprehensive assessment, integrating both the morphological and mechanical evaluations, in order to optimize scaffold design for effective bone-tissue engineering. Nevertheless, further research is required to fully exploit the potential of chitosan- and nanohydroxyapatite-based scaffolds. The authors of this review highlight the significance of the scaffold’s porous structure and morphology for enhancing biocompatibility and nutrient delivery, emphasizing the necessity for the development of new materials that address the complexities of bone defect treatments. It is of the utmost importance to disseminate knowledge on these biomaterials in order to increase interest and application in clinical settings, particularly in the dentistry and surgical fields.

## Figures and Tables

**Table 1:** General characteristics of the selected studies.

Reference	Scaffold Manufacturing Technique	Scaffold Composition	Morphological Characteristics of the Scaffolds	In Vivo Specimen and Utilized Procedure	Conclusions and Clinical Applicability
Jolly et al. [36]	Co-precipitation	B-cyclodextrin/carboxymethyl chitosan/hydroxyapatite fused with date-seed extract (DSE) nanocomposite scaffolds	Nanocomposite scaffolds with tunable size and amendable surface properties with homogenous distribution of needle-shaped particles, moderate hydrophilicity, interconnected rough morphology	In 18 rats, defects were prepared in the skull. The 1st group was the control group, the 2nd group was filled with cerabon (CB), and the 3rd group with the BCHD3 nanocomposite scaffold.	The tested scaffolds were characterized by a porous structure, which is necessary for bone regeneration. BCHD3 with the maximum DSE content may be used in osteogenic tissue engineering due to its bioactivity and cytocompatibility with MG-63 cell lines.
Yin et al. [37]	The preparation of the La-HA nanoparticles by a coprecipitation method and the fabrication of nanohybrid scaffolds by a freeze-drying method	La-doped hydroxyapatite/chitosan (La-HA/CS) biomimetic scaffolds	La-HA nanoparticles have rod-like shapes, and the chemical elements including Ca, P, La, and O were dispersed throughout the La-HA nanoparticles. La-HA nanoparticles represented a monocrystalline structure	Two 5 mm diameter defects were made in the parietal and frontal bones in the rats. The defects were filled with a Ca9La1-HA/CS scaffold on the right side and a control HA/CS scaffold on the left side.	The evaluated scaffolds showed synchronized bone osseointegration and biodegradability. Therefore, they can be considered as a novel and promising platform to facilitate bone remodeling.
Zhao et al. [38]	Uniform CMCS nanofibers were prepared using polyethylene oxide (PEO), while hydroxyapatite (HA)-coated CMCS nanofibers were prepared by biomimetic mineralization method using 5-times simulated body fluid	Carboxymethyl chitosan (CMCS)–hydroxyapatite (HA) composite nanofibers	Electrospun CS and CMCS had good nanofibrous morphology before mineralization; mineral layer was HA crystals. It was concluded that CMCS nanofibers can effectively regulate the nucleation and growth of apatite from the solution	The evaluation of the scaffold in terms of supporting the differentiation of osteogenic bone-marrow stromal cells (mBMSCs) while monitoring alkaline phosphatase activity was performed in rats with skull defects.	Composite nanofibers promoted new bone formation and maturation
Hu et al. [39]	The scaffolds were manufactured using a biomimetic fabrication process based on the emulsion template method and hybrid technology	Nanohydroxyapatite-reinforced hybrid scaffolds loading with icariin (icariin-loaded nHAP/CMCS/PLGA)	The scaffolds formed a hierarchical structure, and the addition of nHAP increased the thickness of the pore walls and the interaction between nHAP molecules and the organic matrix.	Five mm diameter cavities were created in the rat skulls using a low-speed dental drill and sterilized scaffolds were implanted there. Rats were euthanized and evaluated 8 or 12 weeks after implantation.	The inclusion of nHAP and icariin improved the mechanical properties and bioactivity of the material and facilitated the repair of damaged bone tissue. Assessed scaffolds have potential for bone repair and regeneration.
Korpayev et al. [40]	An iterative layering process was developed by combining natural extracellular matrix (ECM) components. Individual layers were prepared using freeze-drying and thermal gelation techniques.	Chitosan/collagen-based biomimetic multi-layered osteochondral scaffolds	The multi-layered scaffold presented a continuous, biomimetic structure. It can be characterized as highly porous material with interconnected pore structure.	Subcutaneous implantation in 6 adult male BALB/c mice and a histological examination were performed after 14 days.	Iterative layering of freeze-dried scaffolds and hydrogel matrixes prepared with ECM components in biomimetic proportions may be a promising strategy without the need for growth factors.
Frohbergh et al. [41]	HA nanoparticles were added to the CTS solution. CTS and CTS-HA were electrospun; 7% CTS was dissolved in TFA and electrocentrifugated. Then, it was stabilized by soaking in NaOH dissolved in ethanol and washed with PBS solution. Scaffolds were cross-linked in 0.1% GP dissolved in PBS.	Genipin-crosslinked chitosan scaffolds (CTS-GP) Genipin crosslinked hydroxyapatite-containing chitosan scaffolds (CTS-HA-GP)	The SEM image shows a network of randomly oriented, bead-free fibers and HA nanoparticles deposited on the surfaces of the fibers.	Two round bone fragments were removed from each side of the mouse skull. One defect was covered with a scaffold, and the other was left as a control. Mice were sacrificed after 1, 2, and 3 months, and the skull was examined for regeneration.	The presence of HA in CTS-GP scaffolds increases their ability to osseointegrate. Scaffolds based on mineralized chitosan cross-linked with genipin may constitute a unique biomaterial with possible clinical significance in the repair of skull bone defects.
Wu et al. [42]	Icariin was dissolved in ethanol and added to a solution of chitosan in 2% acetic acid. Then, Ca(NO_3_)_2_ and KH_2_PO_4_ were added, homogenized, and centrifuged. The obtained solution was placed at 4 °C, then −10 °C, and then lyophilized. After drying, it was soaked in 4% NaOH and deionized water saturated with icariin. Finally, it was frozen and lyophilized.	Icariin and chitosan/hydroxyapatite (icariin-CS/HA)	The icariin–CS/HA composite was characterized by a large number of uniform pores with a diameter of approximately 110 µm.	Radial defects were created in 60 rabbits. All bone tissue and interosseous membrane at the defect site were cleaned and supplemented with icariin–CS/HA, CS/HA scaffolds, or left as a control.	Icariin–CS/HA scaffolds have the ability of osteoconduction and osteoinduction. Due to the low price, simple preparation, and sterilization procedure, icariin–CS/HA scaffolds can be used in bone-tissue engineering.
Huang et al. [43]	HA/Col powder was synthesized by self-assembly of mineralized collagen nanofibers and sterilized by X-rays. Chitosan was dissolved in HCl. Both components were mixed and then the pH was neutralized by adding glycerophosphate.	Chitosan (CS), nanohydroxyapatite (HA), and collagen (Col).	Hydrogel matrix	Eighteen rats were divided into two groups. The first one received only CS/HA/Col gel, and the second one received CS/HA/Col gel with rBMSCs. The solution for the second group was prepared by mixing a pellet containing 5 × 106 newly isolated rBMSCs with 0.5 mL of CS/HA/Col solution.	The CS/HA/Col system can be used as a cell carrier and injected into the body in a minimally invasive manner. Provides a biocompatible environment for cell survival.
Chen et al. [44]	To 40 mL of COL, 60 mL of HCl were added. Then, 2 g of CS were dissolved in the mixture, 6 mL of Ca(NO_3_)_2_, 6 mL of K_2_HPO_4_, 0.2 g of EDC, and 0.125 g of NHS were added. The mixture was frozen at −20 °C and lyophilized. The resulting product was soaked in 5% NaOH, washed with deionized water, and lyophilized.	Chitosan, collagen, and hydroxyapatite	The scaffolds are characterized by an interconnected porous structure. The size of the main pores was approximately 110 μm, and the walls consisted of micropores ranging in size from 271 to 576 nm.	In the radius of 36 rabbits, a bone defect was created without connection with the intra-articular space and the intramedullary canal. A scaffold was placed in the gaps.	Scaffold elements promote the interactions of interfacial bonds, which can initiate repair processes after implantation. This scaffold facilitated endogenous tissue engineering without the need for ex vivo culture of autologous cells, initiates endogenous repair processes in vivo, and effectively facilitates bone repair.
Zhou et al. [45]	PLLA, PLGA, and PCL in a weight ratio of 4:3:3 were dissolved in tetrahydrofuran (THF). The polymer gel was treated with a mixture of ice and water. THF-free scaffolds were obtained by freeze-drying. Similarly, SrHA@PPP scaffolds were prepared by mixing the SrHA solution and polymers before phase separation.	Strontium-substituted hydroxyapatite (SrHA), poly (L-lactic acid) (PLLA), poly (ε-caprolactone) (PCL), and poly (lactic-co-glycolic acid) (PLGA) (SrHA@PPP)	The pore size of the scaffolds was measured to be 115.7 ± 42.1 µm by SEM. SrHA particles were contained in the scaffolds. Many smaller pores are distributed throughout the large pores of the scaffolds, reflecting the hierarchical porous structure.	Eight-week-old male rats were divided into five groups, including a control group. After anesthesia with 3% sodium pentobarbital, 5 mm diameter defects were made on the rats’ skulls, into which scaffold samples were then inserted.	Thanks to the controlled release of BMP-2 and Sr ions from a scaffold loaded with two factors, a synergistic effect on osteogenesis was demonstrated both in vitro and in vivo. Therefore, a biomimetic scaffold would be a promising dual-agent delivery system for the treatment of bone defects.
Zhao et al. [46]	One g of CS was added to 1 mL of acetic acid, and 8.9 g of Col was dissolved in deionized water. Then, solutions of FeCl_2_ H_2_O, FeCl_3_ H_2_O, Ca(NO_3_)_2_, K_2_HPO_4_, and EDC/NHS were added. The mixture was lyophilized; 2.5% NaOH was added to the scaffold and then washed with water until the pH was neutralized. When the CS/Col/Fe_3_O_4_/nHAP became porous, it was lyophilized again.	Chitosan, collagen, nanohydroxyapatite, and Fe_3_O_4_ (CS/Col/Fe3O4/nHAP)	The scaffold was highly porous and exhibited a hierarchical, interconnected, network-like structure, with controllable pore sizes ranging from 100 to 300 µm, which were analogous to natural bone. The surface showed roughness.	Full-thickness defects were made in the skulls of rats. Scaffolds were implanted into the defects. Skin wounds were sutured with biodegradable silk threads	The CS/Col/Fe_3_O_4_/nHAP magnetic scaffold was characterized by excellent bioactivity and osteoinduction, which may be used in the regeneration of bone tissue.
Sun et al. [47]	Three g of chitosan powder was added to 97 g of acetic acid. Then, the Zn-UHANW suspension was added. After mixing, it was frozen at −20 °C and then freeze-dried. Finally, it was immersed in NaOH, rinsed with deionized water and ethanol, and dried.	Chitosan, zinc-containing nanoparticle-decorated ultra-long hydroxyapatite nanowires (Zn-UHANWs/CS)	The scaffolds are highly porous and exhibit a three-dimensional, interconnected pore network with pore sizes ranging from 200 to 400 μm. The surface is rough.	Twenty-four rats were divided into CS, UHANW/CS, and Zn-UHANW/CS scaffold groups. A 3.5 mm diameter and 4 mm depth bone defect was made perpendicularly to the lateral femoral condyle, and the scaffolds were implanted.	Thanks to its biomimetic porous structure, excellent mechanical properties, and good osteogenic activity, the Zn-UHANWs/CS scaffold is a promising candidate for use in the repair of bone defects.
Chen et al. [48]	Two hundred mg of CS-P24 were hydrated in 10 mL of 0.1 M HCl, and HA powder (200 mg) was added with continuous stirring until evenly distributed. Then, the CS-P24 and HA hybrid was freeze-dried at −50 °C and 20 Pa using a 96-well plate as a mold to obtain the CS-P24/HA scaffold.	BMP2-derived peptide, chitosan, hydroxyapatite (CS-P24/HA)	In the SEM image, the porosity of the scaffold was 95.7%. Most pores were between 30 and 90 µm in diameter, and the median volume pore diameter was 59.8 µm.	The rats were divided into three groups. Each had two muscle pouches created on both sides of the back and implanted with CS/HA, CS-5%P24/HA, or CS-10%P24/HA, respectively. Each rat was implanted with two scaffolds of the same type. The animals were sacrificed after 4, 8, and 12 weeks.	CS-P24/HA induced osteogenic differentiation of BMSCs both in vitro and in vivo. CS-P24/HA scaffolds have the potential to be used in bone-tissue engineering.
Yu et al. [49]	CaCl_2_ and CuCl_2_·2H_2_O were dissolved in deionized water, and 15 mL of creatine phosphate were added. The solution was heated at 120 °C. After cooling, it was centrifuged and washed with deionized water and ethanol. Then, it was dried.	Hypoxia-mimicking copper (Cu)-doped mesoporous hydroxyapatite (HAp) microspheres (Cu-MHMs)	The SEM image shows HAp nanorods/nanosheets that have been hierarchically assembled into mesoporous, hollow microspheres of relatively uniform size.	Two 5 mm diameter defects were created in the skulls of 18 rats. The defects were then left empty or implanted with MHM/CS scaffolds or Cu1-MHM/CS scaffolds.	Cu1-MHM/CS scaffolds significantly promote simultaneous new bone formation and neovascularization and have potential for the reconstruction of tissue-engineered vascularized bone.
Xie et al. [50]	HAp/CTS nanocompound was dissolved with 15% Col and 15% PEO in a mixture of 3% HAc and DMSO. Then, electro-spinning was performed. All samples were dried in a vacuum oven.	Hydroxyapatite–collagen–chitosan (HAp/Col/CTS).	In the SEM image, the surfaces of the nanofibers were uneven and contained granules.	Defects were created in the skulls of mice. The acellular scaffolds CTS, HAp/CTS, HAp/Col/CTS, and HAp/Col/CTS with iPSC-MSC nanofibrous stem cells were implanted into the defects, and the control group received no treatment.	Electrospun HAp/Col/CTS nanofibrous scaffolds with osteoinductive activity may be a candidate for directing the osteogenic differentiation of iPSC-MSCs for patient-specific bone-tissue repair and regeneration.
Lai et al. [51]	NaHCO_3_ solution was used to treat Bombyx mori silk, and then sericin was removed with distilled deionized water (DDI). The silk was dissolved in CaCl_2_/CH_3_CH_2_OH/H_2_O, passed through a dialysis membrane, and then freeze-dried. CS and SF were dissolved in a TFA/DCM mixture, and nHAP was added. NMS was immersed in 7% ammonia. CS/SF NMS was first immersed in CaCl_2_, rinsed with DDI, and then immersed in Na_2_HPO_4_.	Chitosan, silk fibroin, nanohydroxyapatite	The nanofibers obtained a “beads on a string” surface morphology with an average fiber diameter of 266 ± 47 nm.	Before implantation, CS/SF/30%nHAP NMS were cultured with hMSCs for 14 days in cell-culture medium. The cell-free and hMSC-free scaffold was implanted into the subcutaneous pocket on both sides of the back of the mouse. The animals were sacrificed after 4 and 8 weeks.	This scaffold is an excellent tool for bone-tissue engineering based on μ-CT and histological analysis.
Jiang et al. [52]	The membrane was mechanically perforated with a pore size of 300 µm and an inter-pore spacing of 1.0 mm. It was then tightly rolled in a concentric manner. The jacket was eroded with acetic acid and attached to the scaffold. A cylindrical scaffold was prepared.	Chitosan (CS), sodium carboxymethyl cellulose (CMC), and nanohydroxyapatite (n-HA)	The scaffold showed a rough surface	The spiral–cylindrical scaffolds were cut into pieces 10 mm long and 3 mm in diameter. A defect was created in the central part of the rabbits’ left radius, filled with a spiral–cylindrical scaffold and secured with sutures. The animals were sacrificed at 4, 8, and 12 weeks after surgery	The manufactured scaffold has the potential to treat large bone defects, and its ability to heal segmental bone defects of critical size is currently being tested.
Liu et al. [53]	HAp/CTS and CTS nanocomposites were dissolved separately in poly(ethylene oxide). Electro-spinning was performed. Then, the samples were dried in a vacuum oven. The scaffolds were prepared by solution casting.	Nanocomposite nanofibrous scaffold of hydroxyapatite–chitosan (nHAp/CTS) seeded with bone-marrow mesenchymal stem cells (BMSCs)	SEM: The surface of the nCTS was smooth, whereas the nHAp/CTS was uneven with a protruding granular morphology. The diameter of nCTS and nHAp/CTS was 200–300 nm.	Two full-thickness defects were created in the skull of 25 rats. nHAp/CTS or nCTS seeded with rat BMSCs consisting of three layers of the scaffold with cells was implanted into the left defect. The scaffold without BMSCs was implanted into the right defect.	nHAp/CTS promotes BMSC adhesion, spreading, and cell viability and proliferation induce osteogenic differentiation of BMSCs by activating integrin and BMP/Smad signaling pathway. nHAp/CTS/BMSCs were superior to nCTS/BMSCs for promoting bone regeneration in vivo.
Kong et al. [54]	The chitosan solution, Ca(NO_3_)_2_ and (NH_4_)2HPO_4_ were frozen in liquid nitrogen and freeze-dried. The outer surface of the freeze-dried tube was covered with a solution of acetic acid and chitosan and dried. After drying, a hollow porous tube with a compact outer chitosan membrane was obtained. A mixture solution of chitosan, Ca(NO_3_)_2_, and (NH_4_)2HPO_4_ was injected. The whole thing was subjected to cryogenic freezing and then freeze-drying.	Multilayer scaffold: chitosan membrane, porous nano-HA–chitosan tube, the porous nano-HA–chitosan core uniform porous scaffold	The layers from outer to inner: compact chitosan membrane (dense, no pores), the porous nano-HA–chitosan tube with a smaller pore size, and the porous nano-HA–chitosan core with a larger pore size. Pore size was quite different in uniform (20–40 µm) and multilayer (100–150 µm) scaffolds, despite similar manufacturing techniques	A 5 mm thick bone defect was created in the fibula bones of 6 rabbits, and then they were divided into two groups: control and study group, which were implanted with multi-layer scaffolds.	The multilayer scaffold was more compatible with respect to cell ingrowth than the uniform porous scaffold. They prevented the formation of fibrous tissue at the defect site, and their central larger pores ensured the supply of nutrients in vivo. The multilayer scaffold supported bone formation.
Li et al. [55]	HA was grafted onto Col by reductive amination. Col and Col/HAs were subjected to self-assembled mineralization and freeze-drying. HA-modified chitosan (HAs/CTS) was prepared by covalent bonding between the carboxyl group of HA and the amino group exposed to CTS. Mineralized powders (Col/HAP, Col/HAs/HAP) and CTS-bound powders (CTS, HAs/CTS) were added to PLGA. The dispersed solutions were frozen and then freeze-dried.	Biomimetic hyaluronic acid oligosaccharides (oHAs)-based composite scaffold	PLGA scaffolds showed a smooth surface with inter-connected pores of random size. Composite scaffolds also showed interconnected pores, but the surface was rough, with a non-agglomerated distribution of particles in the PLGA matrix. Layers of mineral crystals with a structure similar to that observed in bone tissue are present on Col/HA/HAP and Col/oHAs/HAP.	In 10 mice, two subcutaneous pockets were created on each side of the back and filled with scaffolds. The grafts were analyzed histologically and imaged using a light microscope.	The PLGA-based 3D scaffold showed high porosity, water absorption capacity, and a persistent tendency to degrade in the presence of lysozyme. The mechanical strength was similar to that of cancellous bone. In vitro: HA-based scaffolds can facilitate MC3T3-E1 proliferation and differentiation as well as PIEC attachment and proliferation. BMSCs seeded on HA/oHA-based scaffolds showed positive regulation of osteocalcin and ALP protein levels. In vivo: optimal biocompatibility was demonstrated, with abundant cell infiltration, collagen deposition, and biodegradability of the scaffold.

**Table 2:** Detailed characteristics of the included studies.

Reference	Potential Application	Scaffold Characterization Techniques	Presence of Reference Sample	Impact of HA	Type of Used Chitosan
Jolly et al. [36]	Repair of critical size calvarial defects, bone regeneration in general	TEM, water contact angle and SEM, FTIR, X-ray diffraction, compressive strength test	Yes (different concentrations)	Not investigated	Commercial from Sigma Aldrich (St. Louis, MO, USA)
Yin et al. [37]	Bone remodeling	SEM, EDS, XRD, FTIR, compressive strengths—microcomputer controlled electronic universal testing machine, micro-CT	Yes (group of scaffolds was dopped with La)	Not investigated	No info. on origin.
Zhao et al. [38]	Promotion of new bone formation and maturation	SEM, FTIR, XRD, micro-CT	Yes (Carboxymethyl chitosan with or without addition of HA)	Increase of the ALP activity	Commercial from Huamaik Biotechnology Co., Ltd. (Shanghai, China): MW: 2.0 × 10^5^–2.5 × 10^5^, deacetylation Degree ≥90%, degree of substitution of O-carboxymethyl groups: ≥90%
Hu et al. [39]	Accelerated bone regeneration	Field-emission SEM (FESEM), XRD, FTIR, differential scanning calorimetry, thermogravimetric analysis, compression analysis using universal material testing machine	Yes (different combination of concentrations of the scaffolds’ components—icariin-loaded nHA/carboxymethyl chitosan—CMCS/oil-soluble poly(lactide-co-glycolide)—PLGA)	The nHAP and icariin resulted in enhanced mechanical properties and bioactivity	Carboxymethyl chitosan (no additional info.)
Korpayev et al. [40]	Develop multi-layered/multi-component osteochondral mimetic constructs without the need for growth factors	FESEM, micro-CT, Thermo-Haake Modular Advanced Rheometer system (MARS), compression tests	No (but presence of different layers)	Potentially higher elastic modulus	Commercial from Sigma Aldrich (St. Louis, MO, USA), medium MW, DD ≥ 75–85%
Frohbergh et al. [41]	Promotion of regeneration of critical-size craniofacial lesions	Micro-CT and histology (H&E and Mason’s Trichrome)	Yes (comparison of chitosan scaffolds, chitosan scaffolds crosslinked with genipin, and chitosan scaffolds crosslinked with genipin containing hydroxyapatite)	The incorporation of HA into the scaffolds markedly augments their osseointegrative capability	Medium molecular weight chitosan (CTS, 75–85% deacetylated)
Wu et al. [42]	Optical bone repair scaffold for tissue engineering (the filling of bone defect sites and the stimulation of newborn bone tissues)	SEM, HE staining, mechanical properties universal testing machine	Yes (chitosan–hydroxyapatite scaffold with or without addition of icariin)	The release of icariin may be affected by nano-HA particles	Commercial chitosan from Shanghai Bo’ao Biological Technology Co. (Shanghai, China): degree of deacetylation ≥ 90.0%, viscosity < 100 cps, biomedical grade
Huang et al. [43]	A biomimetic gel scaffold that can be injected into body in a minimally invasive manner and provides a biocompatible environment for bone-marrow stem-cell survival	Rheological measurement, pH, and conductivity measurements	Yes (scaffolds with or without rat bone-marrow stem cells)	Not investigated	Commercial from Shandong AK Biotech Ltd. (Shandong, China).
Chen et al. [44]	Facilitated endogenous tissue engineering	XRD, SEM, X-ray photoelectron spectroscopy (XPS), FTIR, compressive strengths using universal material testing machine, liquid displacement method used to test porosity	Yes (control group without a scaffold)	The dimensions and degree of crystallinity of the HA particles facilitate the degradation of the scaffold and the formation of bone tissue following implantation in vivo. Moreover, the distribution of nano-HA provides the geometry and surface topography for the scaffold, which in turn affects cellular behavior	Commercial chitosan from Shanghai Bio Life Science & Technology Co., Ltd. (Shanghai, China)
Zhou et al. [45]	Bone regeneration–biomimetic scaffold as dual-factor delivery system	Transmission electron microscopy (TEM), SEM, EDS, Attenuated total reflectance FTIR, XRD	Yes (different combinations of composition of the scaffolds composed of PLLA/PLGA/PCL with SrHA and BMP-2 protein and polyelectrolytes-modified scaffolds)	The incorporation of trontium-substituted hydroxyapatite SrHA resulted in an enhancement of the mechanical properties of the composite scaffold	Commercial chitosan from Aladdin Industrial Corporation (Shanghai, China): viscosity: 100–200 mPa s, deacetylation ≥ 95%
Zhao et al. [46]	Bone defect repair	SEM, energy-dispersive spectrometry (EDS), FTIR, magnetic property were determined using a physical property measurement system, X-ray photoelectron spectroscopy (XPS), binding energy and junction state of the elements were analyzed	Yes (chitosan–collagen–nanohydroxyapatite scaffold with or without Fe_3_O_4_)	The incorporation of nHAP with a polymer can assist in the elimination of inflammatory processes associated with implant degradation, thereby enhancing the microenvironment at repair sites	Commercial chitosan from Shanghai Bio Science & Technology Co., Ltd. (Shanghai, China)
Sun et al. [47]	Bone defect repair	SEM, TEM, XRD, FTIR	Yes (different types of scaffolds: nanoparticle-decorated ultra-long hydroxyapatite nanowires (UHANWs)–chitosan (UHANWs/CS) porous scaffold and Zn-UHANWs/CS (Zn-UHANWs/CS) porous scaffold	Can significantly enhance the mechanical properties of composite scaffolds	Commercial chitosan from medium viscosity; Aladdin Industrial Corporation (Shanghai, China)
Chen et al. [48]	Repair of bone defects	SEM, mercury intrusion analyzer, XPS	Yes (CS/HA, CS-5% P24/HA, and CS-10% P24/HA; P24-BMP2-derived peptide P24	Not investigated	Commercial chitosan from Sinopham Chemical Reagent Co., Ltd. (Shanghai, China): viscosity: 50–800 mPa·s, degree of de-acetylation: 80–95%
Yu et al. [49]	Reconstruction of vascularized tissue-engineered bone	XRD, SEM, specific surface area, and pore size analysis	Yes (0.2, 0.5 and 1 mol% copper doped mesoporous hydroxyapatite microspheres with or without chitosan)	Composites with HA have high specific surface area, which makes them efficient in drug delivery	Commercial chitosan from Aladdin Industrial Corporation (Shanghai, China): medium viscosity
Xie et al. [50]	Personalized and efficacious bone regeneration	Not executed	Yes (TCP, CTS, HA/CTS)	Not investigated	Commercial chitosan from Sigma Aldrich: derived from crab shells, degree of deacetylation >85%
Lai et al. [51]	Bone regeneration	TGA, XRD, XPS, SEM, NFM, FTIR, TGA, Young’s modulus measurements	Yes (chitosan (CS)–silk fibroin (SF) and cs/sF/nhaP nanofibrous membrane scaffold (NMS))	The incorporation of nHA has been demonstrated to promote osteogenic differentiation of human bone-marrow mesenchymal stem cells. Moreover, the extent of differentiation appears to depend on the concentration of nHA	Commercial chitosan from Fluka Sigma Aldrich (Darmstadt, Germany): deacetylation of 98% and a molecular weight of 1 × 10^5^ Da
Jiang et al. [52]	Bone regeneration	WCA, the swelling behavior, Degradation test, SEM, EDS, weight loss, roasting test	Yes (the hybrid membranes containing 0, 20, 40, and 60 wt % of n-HA)	the 60 wt % n-HA ratio of the scaffold was comparable to that of natural bone, which was beneficial for the integration of the implant into the surrounding bone tissue	Commercial chitosan from Haidebei Marine Bioengineering Co. (Jinan, China): Mw ~200–250 kDa, DD = 95.41%
Liu et al. [53]	Promotion of bone regeneration	X-ray scanning	Yes (comparison of scaffolds composed of nano HA/CS and membranous HA/CS and electrospun nanofibrous chitosan)	Probably HA plays a role in cell adhesion or spreading on nano HA/CTS	No info. on origin of chitosan
Kong et al. [54]	Bone-tissue engineering	SEM, Shimadzu Autograph AG-I testing machine-load of different deformation was measured	Yes (control group without a scaffold)	Not investigated	Commercial chitosan from Haisheng Co. (China): DD = 93.5%, Mη = 1.7 × 10^6^
Li et al. [55]	Bone-tissue engineering	SEM, FTIR, XRD, TGA, TEM, porosity determination, degradability evaluation	Yes (comparison of different scaffolds: poly (lactic-co-glycolic acid), collagen(Col)/HA(hydroxyapatite), COL/HA/CS (chitosan), COL/HAA(hyaluronic acid)/HA/HAA/CTS, COL/oHAs (hyaluronic acid oligosaccharides)/HAoHAs/CS	HA-based scaffolds could facilitate pro-osteoblasts MC3T3-E1 proliferation and differentiation, as well as porcine iliac artery endothelial cell attachment and proliferation	Commercial chitosan from Jinan Haidebei Marine Biological Engineering Co., Ltd. (Jinan, China) 90% deacetylated, Mη ~10^6^

**Table 3:** Quality assessment.

Reference	In Vivo Group Size of Min. 10 Specimens	Presence of Control Group in In Vivo Examination	Detailed Description of Biomaterial Composition	Description of the Effect of Scaffold on the Process of Bone Regeneration	Description of Potential Clinical Applicability of Biomaterial	Total Points	Risk of Bias
Jolly et al. [36]	0	1	1	1	1	4	Low
Yin et al. [37]	1	1	1	1	1	5	Low
Zhao et al. [38]	0	1	1	1	1	4	Low
Hu et al. [39]	0	1	1	1	1	4	Low
Korpayev et al. [40]	0	0	1	1	0	2	Moderate
Frohbergh et al. [41]	0	1	1	1	1	4	Low
Wu et al. [42]	1	1	1	1	1	5	Low
Huang et al. [43]	0	0	1	0	0	1	High
Chen et al. [44].	1	1	1	1	1	5	Low
Zhou et al. [45]	0	1	1	1	1	4	Low
Zhao et al. [46]	0	1	1	1	1	4	Low
Sun et al. [47]	0	1	1	1	1	4	Low
Chen et al. [48]	0	1	1	1	1	4	Low
Yu et al. [49]	0	1	1	1	1	4	Low
Xie et al. [50]	0	1	1	1	1	4	Low
Lai et al. [51]	0	1	1	1	1	4	Low
Jiang et al. [52]	0	1	1	1	1	4	Low
Liu et al. [53]	1	1	1	1	1	5	Low
Kong et al. [54]	0	1	1	1	1	4	Low
Li et al. [55]	0	1	1	0	1	3	Moderate

0—criterion not present in the study. 1—criterion present in the study.
